# Supplementary material for: HIVprotI: an integrated web based platform for prediction and design of HIV proteins inhibitors
Source: J Cheminform. 2018 Mar 9;10:12. doi: 10.1186/s13321-018-0266-y (PMC5845081; doi:10.1186/s13321-018-0266-y)
Supplement: Supplementary file 2 — Additional file 2. Source code of HIVProtI web server. [file 13321_2018_266_MOESM2_ESM.zip › HIVprotI_Source-code/hivprotI/weka-3-6-6/changelogs/CHANGELOG-3-5-1.html]

Weka-3-5-1 Changelog


# Weka-3-5-1 Changelog

Designed for use with Ant.

---

2005-09-30 04:26 eibe

```
Added tests for empty datasets
```

- classifiers/meta/nestedDichotomies/ClassBalancedND.html (1.2)
- classifiers/meta/nestedDichotomies/DataNearBalancedND.html (1.2)
- classifiers/meta/nestedDichotomies/ND.html (1.2)

2005-09-30 04:23 eibe

```
Added tests for empty datasets
```

- classifiers/meta/END.html (1.2)

2005-09-30 03:10 fracpete

```
added hack to remove ResultProducers from the ResultListener list
```

- gui/GenericPropertiesCreator.html (1.6)

2005-09-30 03:09 fracpete

```
tooltip has been disabled in general for now...
```

- gui/visualize/PrintableComponent.html (1.4)

2005-09-30 02:14 mhall

```
branches:  1.1.2;
New icons from Jeffrey
```

- gui/beans/icons/ADTree.gif (1.1)
- gui/beans/icons/ADTree\_animated.gif (1.1)
- gui/beans/icons/AODE.gif (1.1)
- gui/beans/icons/AODE\_animated.gif (1.1)
- gui/beans/icons/BayesNet.gif (1.1)
- gui/beans/icons/BayesNet\_animated.gif (1.1)
- gui/beans/icons/ComplementNaiveBayes.gif (1.1)
- gui/beans/icons/ComplementNaiveBayes\_animated.gif (1.1)
- gui/beans/icons/DecisionStump.gif (1.1)
- gui/beans/icons/DecisionStump\_animated.gif (1.1)
- gui/beans/icons/ID3.gif (1.1)
- gui/beans/icons/ID3\_animated.gif (1.1)
- gui/beans/icons/KStar.gif (1.1)
- gui/beans/icons/KStar\_animated.gif (1.1)
- gui/beans/icons/LBR.gif (1.1)
- gui/beans/icons/LBR\_animated.gif (1.1)
- gui/beans/icons/LMT.gif (1.1)
- gui/beans/icons/LMT\_animated.gif (1.1)
- gui/beans/icons/LWL.gif (1.1)
- gui/beans/icons/LWL\_animated.gif (1.1)
- gui/beans/icons/LeastMedSq.gif (1.1)
- gui/beans/icons/LeastMedSq\_animated.gif (1.1)
- gui/beans/icons/LinearRegression.gif (1.1)
- gui/beans/icons/LinearRegression\_animated.gif (1.1)
- gui/beans/icons/Logistic.gif (1.1)
- gui/beans/icons/Logistic\_animated.gif (1.1)
- gui/beans/icons/M5P.gif (1.1)
- gui/beans/icons/M5P\_animated.gif (1.1)
- gui/beans/icons/MultilayerPerceptron.gif (1.1)
- gui/beans/icons/MultilayerPerceptron\_animated.gif (1.1)
- gui/beans/icons/NBTree.gif (1.1)
- gui/beans/icons/NBTree\_animated.gif (1.1)
- gui/beans/icons/NaiveBayes.gif (1.1)
- gui/beans/icons/NaiveBayesMultinomial.gif (1.1)
- gui/beans/icons/NaiveBayesMultinomial\_animated.gif (1.1)
- gui/beans/icons/NaiveBayesSimple.gif (1.1)
- gui/beans/icons/NaiveBayesSimple\_animated.gif (1.1)
- gui/beans/icons/NaiveBayesUpdateable.gif (1.1)
- gui/beans/icons/NaiveBayesUpdateable\_animated.gif (1.1)
- gui/beans/icons/NaiveBayes\_animated.gif (1.1)
- gui/beans/icons/PaceRegression.gif (1.1)
- gui/beans/icons/PaceRegression\_animated.gif (1.1)
- gui/beans/icons/RBFNetwork.gif (1.1)
- gui/beans/icons/RBFNetwork\_animated.gif (1.1)
- gui/beans/icons/REPTree.gif (1.1)
- gui/beans/icons/REPTree\_animated.gif (1.1)
- gui/beans/icons/RandomForest.gif (1.1)
- gui/beans/icons/RandomForest\_animated.gif (1.1)
- gui/beans/icons/RandomTree.gif (1.1)
- gui/beans/icons/RandomTree\_animated.gif (1.1)
- gui/beans/icons/SMO.gif (1.1)
- gui/beans/icons/SMO\_animated.gif (1.1)
- gui/beans/icons/SMOreg.gif (1.1)
- gui/beans/icons/SMOreg\_animated.gif (1.1)
- gui/beans/icons/SimpleLinearRegression.gif (1.1)
- gui/beans/icons/SimpleLinearRegression\_animated.gif (1.1)
- gui/beans/icons/SimpleLogistic.gif (1.1)
- gui/beans/icons/SimpleLogistic\_animated.gif (1.1)
- gui/beans/icons/UserClassifier.gif (1.1)
- gui/beans/icons/UserClassifier\_animated.gif (1.1)
- gui/beans/icons/VotedPerceptron.gif (1.1)
- gui/beans/icons/VotedPerceptron\_animated.gif (1.1)
- gui/beans/icons/Winnow.gif (1.1)
- gui/beans/icons/Winnow\_animated.gif (1.1)

2005-09-30 02:11 mhall

```
Updated icons thanks to Jeffrey
```

- gui/beans/icons/IB1.gif (1.2)
- gui/beans/icons/IB1\_animated.gif (1.2)
- gui/beans/icons/IBk.gif (1.2)
- gui/beans/icons/IBk\_animated.gif (1.2)
- gui/beans/icons/J48.gif (1.2)
- gui/beans/icons/J48\_animated.gif (1.2)

2005-09-29 02:06 fracpete

```
display of ToolTip for PrintableComponent/Panel now depends on weka/gui/visualize/Visualize.props property "PrintableComponentToolTipShow"
user is asked the first time whether ToolTip is wanted or not
Visualize.props then gets stored in user's home directory
```

- gui/AttributeVisualizationPanel.html (1.22)

2005-09-28 03:18 eibe

```
Added sub-package nestedDichotomies.
```

- gui/GenericPropertiesCreator.props (1.5)

2005-09-27 22:58 fracpete

```
print/println methods are now synchronized, prevents BadLocationExceptions
```

- gui/LogWindow.html (1.3)

2005-09-27 01:30 fracpete

```
added button to enter a perl regular expression for selecting items
```

- gui/ListSelectorDialog.html (1.7)

2005-09-26 06:04 fracpete

```
branches:  1.1.2;
added support for default Experimenter values/setup
```

- gui/experiment/Experimenter.props (1.1)
- gui/experiment/ExperimenterDefaults.html (1.1)

2005-09-26 06:04 fracpete

```
added support for default Experimenter values/setup
```

- gui/experiment/DatasetListPanel.html (1.16)
- gui/experiment/ResultsPanel.html (1.36)
- gui/experiment/SimpleSetupPanel.html (1.10)

2005-09-26 05:33 fracpete

```
removed safeDoubleToString since not used (also exists in DatabaseUtils)
```

- core/converters/DatabaseConnection.html (1.2)

2005-09-26 05:31 fracpete

```
safeDoubleToString returns NaNs now as SQL NULL
```

- experiment/DatabaseUtils.html (1.21)

2005-09-25 05:03 fracpete

```
option -M was missing in getOptions()
```

- filters/unsupervised/instance/RemoveWithValues.html (1.8)

2005-09-22 05:08 eibe

```
Area under ROC is now available for each class from the command-line, the Explorer, the Experimenter, and the KnowledgeFlow.
```

- classifiers/Evaluation.html (1.60)
- experiment/ClassifierSplitEvaluator.html (1.24)
- gui/beans/ClassifierPerformanceEvaluator.html (1.13)
- gui/explorer/ClassifierPanel.html (1.83)

2005-09-22 01:18 fracpete

```
added preamble (GPL etc.)
```

- gui/beans/DataFormatListener.html (1.3)

2005-09-22 01:18 fracpete

```
added preamble (GPL etc.)
```

- classifiers/functions/pace/Matrix.html (1.3)

2005-09-22 01:18 fracpete

```
added preamble (GPL etc.)
```

- classifiers/functions/supportVector/CachedKernel.html (1.4)

2005-09-22 01:18 fracpete

```
added preamble (GPL etc.)
```

- classifiers/bayes/net/estimate/MultiNomialBMAEstimator.html (1.5)

2005-09-22 00:50 fracpete

```
fixed Revision tag
```

- gui/graphvisualizer/BIFFormatException.html (1.3)
- gui/graphvisualizer/BIFParser.html (1.5)
- gui/graphvisualizer/DotParser.html (1.3)
- gui/graphvisualizer/GraphConstants.html (1.4)
- gui/graphvisualizer/GraphEdge.html (1.3)
- gui/graphvisualizer/GraphNode.html (1.3)
- gui/graphvisualizer/HierarchicalBCEngine.html (1.4)
- gui/graphvisualizer/LayoutCompleteEvent.html (1.3)
- gui/graphvisualizer/LayoutCompleteEventListener.html (1.4)
- gui/graphvisualizer/LayoutEngine.html (1.4)

2005-09-22 00:50 fracpete

```
fixed Revision tag
```

- classifiers/misc/FLR.html (1.4)

2005-09-22 00:49 fracpete

```
fixed Revision tag
```

- classifiers/functions/supportVector/Kernel.html (1.3)
- classifiers/functions/supportVector/NormalizedPolyKernel.html (1.3)
- classifiers/functions/supportVector/PolyKernel.html (1.3)
- classifiers/functions/supportVector/RBFKernel.html (1.4)
- classifiers/functions/supportVector/SMOset.html (1.2)

2005-09-22 00:49 fracpete

```
fixed Revision tag
```

- gui/beans/AbstractDataSourceBeanInfo.html (1.2)
- gui/beans/ClassValuePicker.html (1.5)
- gui/beans/ClassValuePickerCustomizer.html (1.4)
- gui/beans/CustomizerClosingListener.html (1.3)
- gui/beans/GraphListener.html (1.3)
- gui/beans/IncrementalClassifierEvent.html (1.6)
- gui/beans/IncrementalClassifierListener.html (1.2)
- gui/beans/LoaderCustomizer.html (1.9)
- gui/beans/SaverCustomizer.html (1.3)
- gui/beans/StripChartCustomizer.html (1.2)
- gui/beans/TrainTestSplitMaker.html (1.6)
- gui/beans/TrainTestSplitMakerCustomizer.html (1.2)
- gui/beans/TrainingSetListener.html (1.3)
- gui/beans/TrainingSetMakerBeanInfo.html (1.2)
- gui/beans/WekaWrapper.html (1.3)

2005-09-22 00:48 fracpete

```
fixed Revision tag
```

- core/BinarySparseInstance.html (1.8)
- core/SelectedTag.html (1.7)
- core/SparseInstance.html (1.16)
- core/Stopwords.html (1.2)
- core/Tag.html (1.6)

2005-09-22 00:48 fracpete

```
fixed Revision tag
```

- gui/DatabaseConnectionDialog.html (1.4)

2005-09-22 00:48 fracpete

```
fixed Revision tag
```

- filters/unsupervised/instance/Normalize.html (1.3)

2005-09-22 00:47 fracpete

```
fixed Revision tag
```

- clusterers/forOPTICSAndDBScan/OPTICS\_GUI/GraphPanel.html (1.2)
- clusterers/forOPTICSAndDBScan/OPTICS\_GUI/OPTICS\_Visualizer.html (1.3)
- clusterers/forOPTICSAndDBScan/OPTICS\_GUI/ResultVectorTableModel.html (1.2)
- clusterers/forOPTICSAndDBScan/OPTICS\_GUI/SERFileFilter.html (1.2)
- clusterers/forOPTICSAndDBScan/OPTICS\_GUI/SERObject.html (1.2)

2005-09-22 00:47 fracpete

```
fixed Revision tag
```

- clusterers/forOPTICSAndDBScan/Databases/Database.html (1.2)
- clusterers/forOPTICSAndDBScan/Databases/SequentialDatabase.html (1.2)

2005-09-22 00:47 fracpete

```
fixed Revision tag
```

- clusterers/DBScan.html (1.2)
- clusterers/OPTICS.html (1.2)

2005-09-22 00:47 fracpete

```
fixed Revision tag
```

- clusterers/forOPTICSAndDBScan/Utils/EpsilonRange\_ListElement.html (1.2)
- clusterers/forOPTICSAndDBScan/Utils/PriorityQueue.html (1.2)
- clusterers/forOPTICSAndDBScan/Utils/PriorityQueueElement.html (1.2)
- clusterers/forOPTICSAndDBScan/Utils/UpdateQueue.html (1.2)
- clusterers/forOPTICSAndDBScan/Utils/UpdateQueueElement.html (1.2)

2005-09-22 00:47 fracpete

```
fixed Revision tag
```

- clusterers/forOPTICSAndDBScan/DataObjects/DataObject.html (1.2)
- clusterers/forOPTICSAndDBScan/DataObjects/EuclidianDataObject.html (1.2)
- clusterers/forOPTICSAndDBScan/DataObjects/ManhattanDataObject.html (1.2)

2005-09-22 00:46 fracpete

```
fixed Revision tag
```

- classifiers/functions/pace/DoubleVector.html (1.3)
- classifiers/functions/pace/ExponentialFormat.html (1.3)
- classifiers/functions/pace/FlexibleDecimalFormat.html (1.3)
- classifiers/functions/pace/FloatingPointFormat.html (1.3)
- classifiers/functions/pace/IntVector.html (1.2)
- classifiers/functions/pace/Maths.html (1.3)

2005-09-22 00:46 fracpete

```
fixed Revision tag
```

- classifiers/bayes/net/search/local/LAGDHillClimber.html (1.3)

2005-09-22 00:46 fracpete

```
fixed Revision tag
```

- classifiers/functions/SMOreg.html (1.8)

2005-09-22 00:46 fracpete

```
fixed Revision tag
```

- classifiers/trees/NBTree.html (1.4)

2005-09-22 00:46 fracpete

```
fixed Revision tag
```

- classifiers/rules/NNge.html (1.3)

2005-09-22 00:45 fracpete

```
fixed Revision tag
```

- associations/tertius/AttributeValueLiteral.html (1.4)
- associations/tertius/Body.html (1.4)
- associations/tertius/Head.html (1.4)
- associations/tertius/IndividualInstance.html (1.3)
- associations/tertius/IndividualInstances.html (1.4)
- associations/tertius/IndividualLiteral.html (1.3)
- associations/tertius/Literal.html (1.3)
- associations/tertius/LiteralSet.html (1.5)
- associations/tertius/Predicate.html (1.3)
- associations/tertius/Rule.html (1.5)
- associations/tertius/SimpleLinkedList.html (1.3)

2005-09-22 00:45 fracpete

```
fixed Revision tag
```

- associations/RuleItem.html (1.2)

2005-09-21 05:21 eibe

```
Replaced computation of the area under the ROC curve. Now uses Wilcoxon-Mann-Whitney statistic instead of trapezoidal method. Results should be identical.
```

- classifiers/evaluation/ThresholdCurve.html (1.19)

2005-09-21 05:10 fracpete

```
fixed Javadoc
```

- filters/unsupervised/instance/RemoveMisclassified.html (1.3)

2005-09-21 05:09 fracpete

```
SPARSE1/2,GAUSSIAN now public
added Revision tag
fixed Javadoc
```

- filters/unsupervised/attribute/RandomProjection.html (1.4)

2005-09-21 05:07 fracpete

```
fixed getOptions (-I instead of -R)
fixed Javadoc
```

- filters/unsupervised/attribute/MathExpression.html (1.3)

2005-09-19 19:45 eibe

```
Fixed problem that could cause stack overflow when attributes had very large values (due to lack of arithmetic precision).
```

- classifiers/trees/j48/BinC45Split.html (1.10)
- classifiers/trees/j48/C45Split.html (1.10)

2005-09-19 01:15 fracpete

```
Liangxiao Jiang fixed the classindex problem (class had to be last attribute)
```

- classifiers/bayes/HNB.html (1.4)

2005-09-18 00:08 fracpete

```
fixed printing of options
```

- classifiers/bayes/net/BayesNetGenerator.html (1.6)

2005-09-17 04:16 fracpete

```
fixed check for zero training instances (used wrong dataset)
```

- classifiers/trees/LMT.html (1.3)

2005-09-17 04:16 fracpete

```
now checks for zero training instances
```

- classifiers/meta/RandomCommittee.html (1.7)

2005-09-17 04:15 fracpete

```
now checks for numeric class
```

- classifiers/meta/AdaBoostM1.html (1.27)

2005-09-17 04:14 fracpete

```
now checks for numeric class and string attributes in the correct order
```

- classifiers/lazy/LBR.html (1.6)

2005-09-17 04:13 fracpete

```
now checks for numeric class
```

- classifiers/lazy/IB1.html (1.14)

2005-09-17 04:12 fracpete

```
removed try-catch-block that blocked an exception necessary for testing
```

- classifiers/functions/PaceRegression.html (1.2)

2005-09-17 04:11 fracpete

```
now deletes all instances with missing class first
```

- classifiers/functions/LeastMedSq.html (1.10)

2005-09-17 04:10 fracpete

```
now checks first whether class is numeric
```

- classifiers/bayes/AODE.html (1.11)

2005-09-17 01:53 fracpete

```
added support for string attributes
all check methods return now a boolean array of length 2 (0=success yes/no, 1=acceptable yes/no)
all System.out.print/println are redirected to internal print/println, in order to suppress output in silent mode
number of instances in test/train sets can now be specified (some classifiers need more instances)
```

- classifiers/CheckClassifier.html (1.17)

2005-09-14 21:41 fracpete

```
changed private members/methods to protected
```

- classifiers/bayes/NaiveBayesSimple.html (1.15)

2005-09-14 06:30 fracpete

```
RANDOMIZED input order not yet implemented, therefore always set to ORDERED
```

- datagenerators/clusterers/BIRCHCluster.html (1.2)

2005-09-13 23:47 fracpete

```
Ashraf fixed a small bug relating to the white background of
plot labels (rather than the background color of the panel), occurring
in JDK1.4 and above
```

- gui/visualize/MatrixPanel.html (1.12)

2005-09-13 21:55 fracpete

```
added Generate button for datagenerators support
```

- gui/explorer/PreprocessPanel.html (1.54)

2005-09-13 21:54 fracpete

```
panel for datagenerators
```

- gui/explorer/DataGeneratorPanel.html (1.1)

2005-09-13 21:43 fracpete

```
added datagenerators and clusterdefinitions
```

- gui/GenericObjectEditor.props (1.110)

2005-09-13 21:24 fracpete

```
increased the scrollbar increment from 1 to 20, for better mouse wheel support
```

- gui/GenericObjectEditor.html (1.44)

2005-09-13 21:23 fracpete

```
added Edit button
```

- gui/GenericArrayEditor.html (1.14)

2005-09-13 21:18 fracpete

```
new structure for data generators
```

- datagenerators/classifiers/regression/Expression.html (1.1)
- datagenerators/classifiers/regression/MexicanHat.html (1.1)

2005-09-13 21:18 fracpete

```
new structure for data generators
```

- datagenerators/clusterers/SubspaceCluster.html (1.1)
- datagenerators/clusterers/SubspaceClusterDefinition.html (1.1)

2005-09-13 21:17 fracpete

```
new structure for data generators
```

- datagenerators/ClassificationGenerator.html (1.1)
- datagenerators/ClusterDefinition.html (1.1)
- datagenerators/ClusterGenerator.html (1.3)
- datagenerators/DataGenerator.html (1.1)
- datagenerators/RegressionGenerator.html (1.1)
- datagenerators/Test.html (1.2)

2005-09-13 21:17 fracpete

```
new structure for data generators
```

- datagenerators/classifiers/classification/Agrawal.html (1.1)
- datagenerators/classifiers/classification/BayesNet.html (1.1)
- datagenerators/classifiers/classification/LED24.html (1.1)
- datagenerators/classifiers/classification/RDG1.html (1.1)
- datagenerators/classifiers/classification/RandomRBF.html (1.1)

2005-09-13 21:16 fracpete

```
removed due to new structure
```

- datagenerators/BIRCHCluster.html (1.4)
- datagenerators/Generator.html (1.3)
- datagenerators/RDG1.html (1.4)

2005-09-12 23:42 fracpete

```
added paper references to Javadoc and globalInfo
```

- classifiers/trees/m5/M5Base.html (1.11)

2005-09-12 23:39 fracpete

```
fixed javadoc
```

- classifiers/rules/M5Rules.html (1.4)

2005-09-12 23:38 fracpete

```
added paper references to Javadoc
```

- classifiers/trees/M5P.html (1.2)

2005-09-08 23:42 eibe

```
REPTree can now deal with datasets that have no attributes other than the class attribute. That is necessary for the wrapper attribute selection technique to work. In that case it simply uses ZeroR.
```

- classifiers/trees/REPTree.html (1.21)

2005-08-23 05:01 fracpete

```
meta classifier divides the training data into equally sized chunks and feeds those to copies of the base classifier
it uses Vote, based on all previously generated base classifiers, for predictions
```

- classifiers/meta/Dagging.html (1.1)

2005-08-21 07:22 fracpete

```
classes like weka.core.Instances expect fixed order of arguments from commandline, therefore the first two empty strings have to be removed
```

- gui/SimpleCLI.html (1.7)

2005-08-18 03:41 fracpete

```
the scrollbar increments are now customizable
the layout dimensions can be defined (default 1024x768)
preferred layout extension definable (default .kf)
user components can be stored in XML (default no)
the connection to and fro MetaBeans from the user toolbar still pointed to the old location, the MetaBean was added to the toolbar - positions are now updated, as soon as MetaBean is placed in layout
```

- gui/beans/KnowledgeFlowApp.html (1.13)

2005-08-18 03:38 fracpete

```
fixed java warning
```

- core/xml/XMLSerialization.html (1.9)

2005-08-18 03:37 fracpete

```
the scrollbar increments are now customizable
the layout dimensions can be defined (default 1024x768)
preferred layout extension definable (default .kf)
user components can be stored in XML (default no)
```

- gui/beans/Beans.props (1.13)

2005-08-18 03:31 fracpete

```
added support for UserToolBar
```

- gui/beans/xml/XMLBeans.html (1.5)

2005-08-16 00:55 mhall

```
Fixed bug that prevented polyline and polygon selection options from working with single button mice. The Alt key can now be held down to simulate button 2
```

- gui/visualize/VisualizePanel.html (1.24)

2005-08-15 02:35 eibe

```
Forgot to delete printStackTrace() statement.
```

- classifiers/meta/Grading.html (1.7)

2005-08-09 00:12 mhall

```
Fixed a bug in adding to the user tool bar
```

- gui/beans/MetaBean.html (1.6)

2005-08-04 23:49 fracpete

```
updated classname in Exception: LogitBoost -> RacedIncrementalLogitBoost
```

- classifiers/meta/RacedIncrementalLogitBoost.html (1.6)

2005-08-04 23:48 fracpete

```
removed restriction on string atts
```

- classifiers/meta/CVParameterSelection.html (1.30)

2005-08-04 23:47 fracpete

```
removed restriction on string atts
```

- classifiers/meta/Bagging.html (1.32)

2005-08-02 03:05 fracpete

```
added "Close" button, which is displayed if parent frame is set
```

- gui/SetInstancesPanel.html (1.8)

2005-08-02 02:53 fracpete

```
changed option -matlab into more intuitive -cost-matrix
```

- classifiers/meta/CostSensitiveClassifier.html (1.22)
- classifiers/meta/MetaCost.html (1.17)

2005-08-01 06:03 kurtd

```
Adding the new Gaussian Processes classifier
```

- classifiers/IntervalEstimator.html (1.1)
- classifiers/functions/GaussianProcesses.html (1.1)

2005-08-01 04:17 fracpete

```
minor clean-up, after XMLSerialization got upgraded
```

- experiment/xml/XMLExperiment.html (1.4)

2005-08-01 04:16 fracpete

```
added support for Matrix (new+old) and CostMatrix
```

- core/xml/XMLBasicSerialization.html (1.4)

2005-08-01 04:14 fracpete

```
added output in and parsing of Matlab notation (one line notation)
```

- core/Matrix.html (1.22)

2005-08-01 04:13 fracpete

```
added output in and parsing of Matlab notation (one line notation)
```

- core/matrix/Matrix.html (1.3)

2005-08-01 04:12 fracpete

```
getArrayDimensions now more general
```

- core/Utils.html (1.48)

2005-07-29 01:33 mhall

```
Adjusted the size of the KnowledgeFlow window
```

- gui/GUIChooser.html (1.24)

2005-07-29 01:32 mhall

```
Now provides a mechanism to add a MetaBean to a user tab in the tool bar. User components are saved on exit and loaded on start up.
```

- gui/beans/BeanVisual.html (1.7)

2005-07-27 02:50 fracpete

```
Experimenter generated Exception with the toString() method, while adding the classifier
```

- classifiers/meta/MultiBoostAB.html (1.7)

2005-07-22 01:17 mhall

```
Nicer to use now
```

- gui/explorer/AttributeSelectionPanel.html (1.33)

2005-07-22 00:01 mhall

```
Is now a WeightedInstancesHandler
```

- classifiers/meta/AttributeSelectedClassifier.html (1.19)

2005-07-13 06:39 fracpete

```
fix for XML serialization: setRefreshWidth() only if getGraphics() not null
```

- gui/beans/StripChart.html (1.12)

2005-07-13 06:32 fracpete

```
for XML serialization: made get methods for source/target public,  added get method for eventname
```

- gui/beans/BeanConnection.html (1.4)

2005-07-13 06:22 fracpete

```
command line options now state that you can load normal and XML setup files with -l option and save with -s, depending on extension
```

- experiment/Experiment.html (1.23)

2005-07-13 06:19 fracpete

```
cleaned up imports
```

- classifiers/xml/XMLClassifier.html (1.5)

2005-07-13 06:16 fracpete

```
register-method automatically adds read/write method for class
```

- core/xml/XMLSerializationMethodHandler.html (1.3)

2005-07-13 06:10 fracpete

```
fixed ignored handling
```

- core/xml/PropertyHandler.html (1.3)

2005-07-13 03:59 mhall

```
Fixed bug in field width setting for merit output
```

- attributeSelection/AttributeSelection.html (1.38)

2005-07-12 22:02 fracpete

```
removed testEigen(...) call (was commented out)
```

- attributeSelection/PrincipalComponents.html (1.28)

2005-07-12 21:58 fracpete

```
branches:  1.2.2;
added toString() method
```

- core/matrix/LinearRegression.html (1.2)

2005-07-11 23:51 fracpete

```
clone() no longer throws a CloneNotSupportedException
due to changes of the superclass weka.core.Matrix
```

- classifiers/evaluation/ConfusionMatrix.html (1.6)

2005-07-11 23:12 fracpete

```
branches:  1.1.2;
matrix sub-package replaces the old weka.core.Matrix class
based on JAMA
```

- core/matrix/CholeskyDecomposition.html (1.1)
- core/matrix/EigenvalueDecomposition.html (1.1)
- core/matrix/LUDecomposition.html (1.1)
- core/matrix/Maths.html (1.1)
- core/matrix/QRDecomposition.html (1.1)
- core/matrix/SingularValueDecomposition.html (1.1)

2005-07-10 00:36 fracpete

```
changed "enum" into "enm" to make it Java 1.5 compatible
```

- core/Attribute.html (1.35)

2005-07-08 07:02 eibe

```
Introduced relation-valued attributes.
```

- core/Instance.html (1.21)
- core/Instances.html (1.61)

2005-07-08 02:48 mhall

```
Initial import
```

- gui/beans/Associator.html (1.1)
- gui/beans/AssociatorBeanInfo.html (1.1)
- gui/beans/AssociatorCustomizer.html (1.1)

2005-07-08 02:48 mhall

```
branches:  1.1.2;
Initial import
```

- gui/beans/icons/DefaultAssociator.gif (1.1)
- gui/beans/icons/DefaultAssociator\_animated.gif (1.1)

2005-07-07 04:20 mhall

```
Changed getRanges() to use a StringBuffer instead of string concatenation
```

- core/Range.html (1.15)

2005-07-05 04:15 mhall

```
Fixed bug in event constraints that prevented a BatchClassifier event from being generatable in the case where a classifier has already been trained and there is only an incoming testSet connection
```

- gui/beans/Classifier.html (1.20)

2005-07-04 03:14 mhall

```
Changed so that the link in the docs now points to the ExplorerGuide
```

- build.xml (1.26)

2005-07-04 03:12 eibe

```
Changed year and URL in LogPanel.
```

- gui/explorer/Explorer.html (1.33)

2005-07-04 00:56 eibe

```
FilteredClassifier now extends SingleClassifierEnhancer. Note that this means that the double hyphen construct now needs to be used to specify the classifier options. Another change: Discretize is now the default filter.
```

- classifiers/meta/FilteredClassifier.html (1.21)

2005-07-04 00:54 eibe

```
Fixed some bugs in handling of option setting errors.
```

- classifiers/SingleClassifierEnhancer.html (1.3)

2005-07-04 00:04 mhall

```
Now extends the appropriate base class
```

- classifiers/meta/OrdinalClassClassifier.html (1.12)

2005-07-03 23:59 eibe

```
Decorate now extends RandomizableIteratedSingleClassifierEnhancer for simplified option handling. Unfortunately, the letters for the command line options had to be changed. What used to be I is now E, and what used to be M is now I. Also, the maximum and desired number of iterations are now both set to 10 by default (instead of 50 and 15 respectively).
```

- classifiers/meta/Decorate.html (1.4)

2005-07-03 23:16 eibe

```
Now allows random codes with fewer columns than classes (handy for datasets with lots of classes). Now implements RandomizableSingleClassifierEnhancer for easier option handling.
```

- classifiers/meta/MultiClassClassifier.html (1.39)

2005-07-03 22:55 eibe

```
Changed letter for command line option speciyfing tolerance parameter as it conflicted with the option for specifying a test set.
```

- classifiers/functions/SMO.html (1.55)

2005-07-03 22:50 eibe

```
Now implements RandomizableSingleClassifierEnhancer to simplify option handling.
```

- classifiers/meta/ThresholdSelector.html (1.32)
